# Alcohol consumption, alcohol dependence, and related mortality in Italy in 2004: effects of treatment-based interventions on alcohol dependence

**DOI:** 10.1186/1747-597X-8-21

**Published:** 2013-06-13

**Authors:** Kevin D Shield, Jürgen Rehm, Gerrit Gmel, Maximilien X Rehm, Allaman Allamani

**Affiliations:** 1 Centre for Addiction and Mental Health (CAMH), Toronto, Canada; 2 Institute of Medical Science, University of Toronto, Toronto, Canada; 3 Institute for Clinical Psychology and Psychotherapy, TU Dresden, Germany; 4 Dalla Lana School of Public Health (DLSPH), University of Toronto, Toronto, Canada; 5 Department of Psychiatry, University of Toronto, Toronto, Canada; 6 Ecole Polytechnique Fédérale de Lausanne, Lausanne, Switzerland; 7 Faculty of Arts and Sciences/Politics and Governance, Ryerson University, Toronto, Canada; 8 Region of Tuscany Health Agency, Florence, Italy

**Keywords:** Alcohol consumption, Heavy drinking, Alcohol dependence, Alcohol dependence treatment, Italy, Europe, Mortality, Burden of disease

## Abstract

**Background:**

The tradition of consuming alcohol has long been a part of Italian culture and is responsible for a large health burden. This burden may be reduced with effective interventions, one of the more important of which is treatment for Alcohol Dependence (AD). The aim of this article is to estimate the burden of disease in Italy attributable to alcohol consumption, heavy alcohol consumption, and AD. An additional aim of this paper is to examine the effects of increasing the coverage of treatment for AD on the alcohol-attributable burden of disease.

**Methods:**

Alcohol-attributable deaths and the effects of treatments for AD were estimated using alcohol-attributable fractions and simulations. Deaths, potential years of life lost, years lived with disability, and disability adjusted life years lost were obtained for 2004 for Italy and for the European Union from the Global Burden of Disease study. Alcohol consumption data were obtained from the Global Information System on Alcohol and Health. The prevalences of current drinkers, former drinkers, and lifetime abstainers were obtained from the GENder Alcohol and Culture International Study. The prevalence of AD was obtained from the World Mental Health Survey. Alcohol relative risks were obtained from various meta-analyses.

**Results:**

5,320 deaths (1,530 female deaths; 3,790 male deaths) or 5.9% of all deaths (4.9% of all female deaths; 6.3% of all male deaths) of people 15 to 64 years of age were estimated to be alcohol-attributable. Of these deaths, 74.5% (61.3% for females; 79.8% for males) were attributable to heavy drinking, and 26.9% (25.6% for females; 27.5% for males) were attributable to AD. Increasing pharmacological AD treatment coverage to 40% would result in an estimated reduction of 3.3% (50 deaths/year) of all female and 7.6% (287 deaths/year) of all male alcohol-attributable deaths.

**Conclusions:**

Alcohol was responsible for a large proportion of the burden of disease in Italy in 2004. Increasing treatment coverage for AD in Italy could reduce that country’s alcohol-attributable burden of disease.

## Introduction

Alcohol consumption plays a large traditional role in all European countries; however, not all countries (and regions) have similar drinking cultures and drinking trends. In contrast to other regions in Europe that have more detrimental patterns of alcohol consumption, Italy has a Mediterranean drinking culture, which features regular consumption of alcohol with a large prevalence of daily drinkers who often consume alcohol with meals. Italian culture also has a high tolerance of the social outcomes of drinking, but has no tolerance for public drunkenness [1,2].

One of the most severe health consequences of alcohol consumption is Alcohol Dependence (AD). The Diagnostic and Statistical Manual of Mental Disorders (version IV) (DSM IV) defines AD as a maladaptive use of alcohol with clinically significant impairment as manifested by at least three of the following criteria within any one year period: tolerance; withdrawal; taken in greater amounts or over a longer time course than intended; desire or unsuccessful attempts to cut down or control use; social, occupational or recreational activities given up or reduced; and/or continued use despite knowledge of physical or psychological consequences of that use [3]. In 2009 65,310 people with AD attended an alcohol treatment program in Italy The women/men ratio was 1: 3.7 [4]. Overall, the proportion of people with AD in treatment in 2009 was less than 10%, based on AD prevalence estimates for Italy published by Scafato and colleagues [5]. The proportion of people with AD who are being treated for AD is very similar to the European average [2].

The number of services in Italy for the treatment of AD (which are presently primarily community services) has increased from 280 in 1996 to 514 in 2009 [4]. Treatment for AD in Italy is based more on a psychosocial or an educational approach than on pharmacological treatment; pharmacological treatment was used in only 28.8% of treatment cases in 2009 [4]. Prescribed drugs were sodium oxibate (gamma hydroxybutyrate), metadoxine, disulfiram, and chlordiazepoxide, and occasionally naltrexone and acamprosate. During 2001 to 2009 counselling was used in 26.5% of AD treatment cases, psychosocial advice (14.5%), self-help groups (Alcoholics Anonymous, 7%), inpatient short treatment (5%), and therapeutic community (2.7%). Collaboration between alcohol addiction services and self-help groups [4], namely Alcoholics Anonymous (AA) or Club of Treated Alcoholics (CAT) [6], is common, affecting 50% of cases, even if such addiction services refer formally to these groups in only 7% of cases.

Given that the coverage of treatment for AD in Italy is currently estimated to be less than 10%, the burden of alcohol consumption in Italy may be reduced if coverage for AD in Italy were increased. Accordingly, the aim of this article is to calculate for Italy for 2004 the alcohol-attributable burden of disease caused by alcohol consumption, heavy alcohol consumption, and AD, and to estimate the effects of increasing the coverage of AD treatments on the alcohol-attributable burden of disease.

## Methods

### Alcohol consumption data

Data for *per capita* consumption of alcohol were obtained from a government survey of the World Health Organization and the Global Information System on Alcohol and Health [7,8]. Data for the prevalence of current drinkers (people who have consumed at least one standard drink of alcohol in the past year), former drinkers (people who have consumed at least one standard drink of alcohol, but did not do so in the past year), and lifetime abstainers (people who have never consumed at least one standard drink) were obtained from the GENder Alcohol and Culture International Study (GENACIS) survey [9,10]. The Italian GENACIS survey was a national telephone survey conducted in 2000 which sampled a total of 1000 people (514 women and 486 men).

To correct for the undercoverage of alcohol consumption measured in surveys [11], we triangulated the survey data with data on *per capita* consumption using methods published by Rehm and colleagues (see [12,13] for full details of the mathematical models). For this method, we used 80% of adult *per capita* consumption when modelling alcohol consumption in order to account for alcohol not consumed and for undercoverage present in observational studies that the Relative Risks (RRs) used in the meta-analyses are based upon [12,13]. This triangulation method assumes that the alcohol consumption distribution will follow a Gamma distribution [12,13]. The first step in this method is the calculation of sex- and age-specific mean alcohol consumption using population level alcohol consumption data (obtained from *per capita* consumption estimates), data on the prevalence of current drinkers (obtained from population surveys), and the sex and age relative amounts of alcohol consumed (obtained from population surveys). The second step is the calculation of the scale parameter of the alcohol consumption Gamma distribution for each age and sex group. The scale parameter is calculated using the association between the mean alcohol consumption and the variance of a population level alcohol consumption Gamma distribution (as described by Kehoe and colleagues [12]).

### Patterns of alcohol consumption

The prevalence of binge alcohol consumption for 2009 (5 or more drinks on one occasion for men (60 grams of pure alcohol or more) and 4 or more standard drinks on one occasion for women (48 grams of alcohol or more)) was based on the pattern of drinking score [14]. The prevalence of irregular binge drinking for 2009 was obtained from a 2010 European Commission report [15]. In the European Commission report, the prevalence of binge alcohol consumption was defined as consuming more than 5 alcoholic drinks per occasion.

Heavy alcohol consumption was defined as consuming on average more than 60 grams of alcohol per day for men (the equivalent of consuming 5 drinks or more) and more than 40 grams of alcohol per day for women (the equivalent of consuming 3 and 1/3 standard drinks or more).

The pattern in which alcohol is consumed at the population level is best summarized by the metric known as the pattern of drinking score, which is a composite measure assessing the manner in which individuals consume alcohol, and which was developed as part of the Comparative Risk Assessment study within the Global Burden of Disease (GBD) study [16,17]. The 2005 pattern of drinking score for Italy was obtained from the 2005 GBD study.

### Data on alcohol dependence

Data on AD for Italy was obtained from a publication authored by Scafato and colleagues [5]. Additional data on AD for Italy, Southern Europe, and the European Union (EU) were obtained from the World Mental Health Survey (ESEMeD/MHEDEA 2000 Investigators, 2004; [18]). These data likely underestimate the true population prevalence of AD, as questions used to measure AD in the World Mental Health Survey were only asked of those respondents who scored positively on questions relating to alcohol abuse [19]. To correct for this undercoverage, data from the World Mental Health Survey were combined with data from the German Mental Health Survey, where both dependence and abuse were assessed independently [20,21]. Sensitivity analyses were performed using AD prevalence estimates data from Lepine and colleagues [22]. These estimates of AD are from the same source as for the main analysis, but were not corrected for undercoverage. Additionally, sensitivity analyses were performed using the corrected estimates from the World Mental Health Survey for southern Europe (Cyprus, Greece, Italy, Malta, Portugal and Spain) and the EU.

### Modelling alcohol-attributable mortality and morbidity

To calculate the alcohol-attributable burden of disease, we utilized Alcohol-Attributable Fractions (AAFs), where an AAF is defined as the percentage of mortality that would not occur if everyone was a lifetime abstainer. For diseases and conditions which are entirely attributable to alcohol consumption (i.e. where alcohol is a necessary cause in the development of the disease or condition, such as AD), the AAF is equal to 1; however, for alcohol-related diseases, conditions and injuries where alcohol is not a necessary cause, we calculated the AAFs by combining exposure indicators and RR functions. (See [2] for the AAF calculation methodology for total alcohol consumption, heavy alcohol consumption, and AD, and see Additional file 1 for the sources of all RR functions for each disease modelled). It should be noted that the number of deaths due to heavy alcohol consumption and AD overlap as most people with AD are also heavy consumers of alcohol.

### Modelling the effects of treatment for alcohol dependence

To estimate the effects of increasing AD treatment rates on alcohol-attributable mortality, we simulated the effects of an increase in the coverage of AD treatment, assuming 40% of all people with AD were receiving treatment. For these simulations, we examined the effects of increasing the AD treatment coverage for five different treatments: Acamprosate and opioid antagonists therapy (referred to in this paper as pharmacological treatment) [23,24], Motivational Interviewing/Cognitive Behavioral Therapy (MI/CBT) [25,26], MI/CBT assuming the upper level of effectiveness, and Brief Interventions (BI) (in two different hospital settings) [27]. Additional file 2 outlines the assumptions used when modelling the effects of AD treatments on alcohol consumption and mortality (see also [2,28]).

For our simulation model, we generated 100,000 data points or “drinkers” to represent the drinking population of Italy using the Gamma distribution as described above. The data points that were identified as drinkers with AD were selected randomly among people who drink more heavily (men who consume 72 grams or more of pure alcohol per day and women who consume 48 grams or more of pure alcohol per day), with the total number of data points selected being equal to the number of people in Italy with AD among every 100,000 drinkers. As the effect of treatment interventions can be translated into a shift in average alcohol consumption or as a decrease in the RR of mortality, the effects of interventions were calculated using AAFs. The AAFs were evaluated before and after interventions and the differences between the estimates were used to calculate the deaths prevented for each given increase in treatment coverage.

The number of alcohol-attributable deaths was calculated by sex according to the following formula:

$$\mathrm{Mortality}=\sum_{a}^{n\_a}\left[\mathrm{Mortalit}y_{\mathrm{cause}\_a}*\left(\frac{\sum_{b}^{N\_b}RR_{\mathrm{Norm}\_a}{\left(x\right)}_{\mathrm{Norm}\_b}+\sum_{c}^{N\_c}RR_{\mathrm{AD}\_a}{\left(x\right)}_{\mathrm{AD}\_c}-1}{\sum_{b}^{N\_b}RR_{\mathrm{Norm}\_a}{\left(x\right)}_{\mathrm{Norm}\_b}+\sum_{c}^{N\_c}RR_{\mathrm{AD}\_a}{\left(x\right)}_{\mathrm{AD}\_c}}\right)\right]$$

where “cause_a” represents a given alcohol-related cause of death; and “n_a” represents the total number of alcohol-related diseases for which the alcohol-attributable mortality was calculated (see Additional file 2). “Mortality_cause_a_” represents the mortality for cause “a”; “N_b” represents the number of people who do not have AD (“b”); and “N_c” represents the number of people who are alcohol dependent (“c”). “RR_Norm_a_(x)_Norm_b_” represents the RR for the alcohol-related disease or condition “a” given an average daily alcohol consumption of x for person “b”; and “RR_AD_a_(x)_AD_c_” represents the RR for the alcohol-related disease or condition “a” given an average daily alcohol consumption of x for person “c”. For people with AD, the RR function was estimated by multiplying the general population RR by 2 to account for the overall higher mortality risk of people with AD [29].

### Mortality and morbidity data

Data on the number of deaths, Potential Years of Life Lost (PYLL), Years Lived with Disability (YLD), and Disability Adjusted Years of Life (DALYs) lost by cause for 2004 were obtained from the 2004 GBD Study [30].

### Population data

Data on population estimates for Italy and for other European countries for 2005 were obtained from the 2005 GBD study [31].

### Software

All analyses were performed using R version 2.13.0 [32] and Mathematica version 8 [33].

### Ethics

As this analysis was performed on data already collected ethics approval was not required from the Centre for addiction and Mental Health. During the original studies (which this analysis is based on) written informed consent was obtained from participants for publication reports based on secondary data analysis.

## Results

### Alcohol consumption in Italy

**Figure 1:**
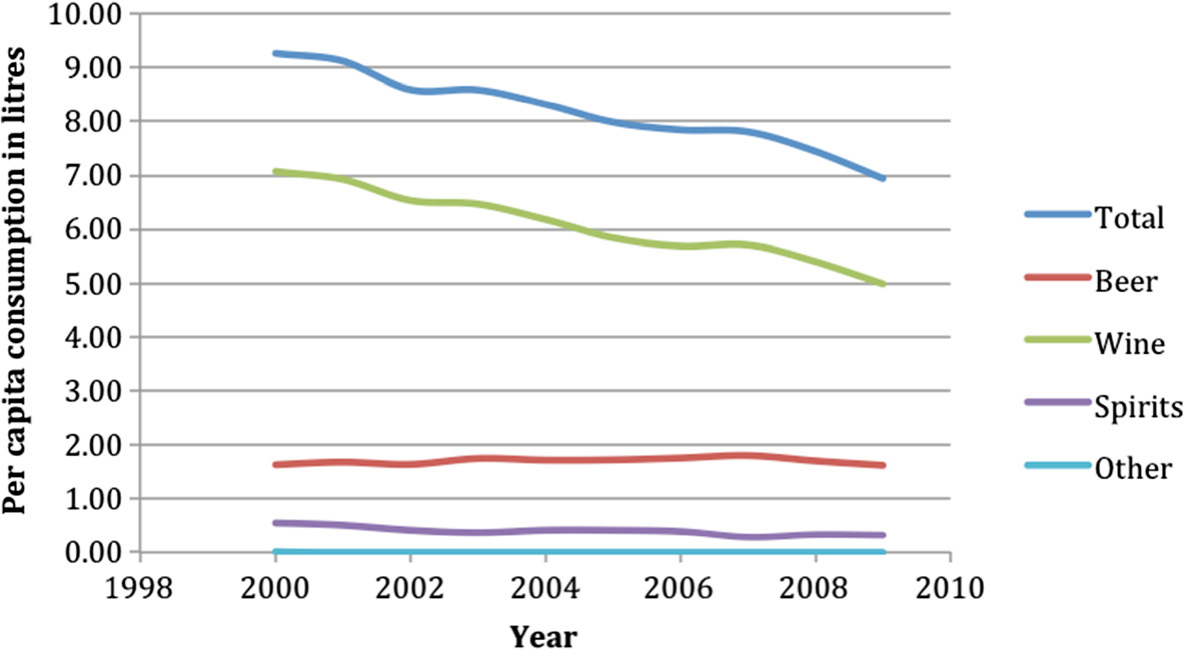
**Trends in recorded alcohol consumption in Italy since 2000 (data obtained from the Global Information System on Alcohol and Health **[7,8]**).**

Italy’s adult *per capita* consumption in 2009 was 9.6 litres (l) of pure alcohol (2.4 l unrecorded consumption; 7.2 l recorded consumption), which was significantly lower than the EU average of 12.5 l of pure alcohol. Of the 7.2 l of recorded consumption, wine was by far the most frequently consumed beverage, constituting 73% of all recorded alcohol consumed. Beer was the second most frequently consumed alcoholic beverage, constituting 22% of all recorded alcohol consumed, and spirits were the third most frequently consumed alcoholic beverage, constituting 5% of all recorded alcohol consumed. Figure 1 illustrates trends in recorded alcohol consumption in Italy from 2000 to 2009 by beverage type (based on 3-year averages with the consumption in a given year calculated by averaging the recorded adult *per capita* consumption that year, the following year and the previous year). Recorded adult *per capita* alcohol consumption decreased from 2000 to 2009, dropping from just over 9 l of pure alcohol in 2000 to just over 7 l of pure alcohol in 2009, which can be attributed to a decrease in recorded wine consumption (see Additional file 3).

Italy had a pattern of drinking score of 1 in 2009 (the least detrimental score possible). For people aged 15 to 64 years of age in Italy in 2009, an estimated 4.6% of men and 9.9% of women were lifetime abstainers, 3.1% of men and 4.4% of women were former drinkers, and 92.3% of men and 85.7% of women were current drinkers. Among current drinkers, 12.4% of male current drinkers and 7.7% of female current drinkers were considered to be heavy consumers of alcohol. In 2009 the prevalence of irregular binge drinking in Italy was estimated to be 14% for binge drinking approximately once a week and was estimated to be 8% for binge drinking approximately once a month.

### Alcohol dependence in Italy

Scafato and colleagues estimated the prevalence of AD in Italy to be 0.9% for women and 3.1% for men. This prevalence estimate corresponds to 165,000 women and 612,000 men aged 15 to 64 years of age. Using data from the ESEMeD study (corrected for underestimation), we estimated that 93,600 women and 149,800 men 18 to 64 years of age in Italy have AD, which corresponds to a prevalence estimate for AD of 0.5% of all women and 0.8% of all men. For comparison purposes, using corrected data from the ‘ study, we estimated that the prevalence of AD for Southern Europe was 0.6% for women and 1.7% for men, and that the prevalence of AD for the EU was 1.3% for women and 4.8% for men.

### Alcohol-attributable harms

**Figure 2:**
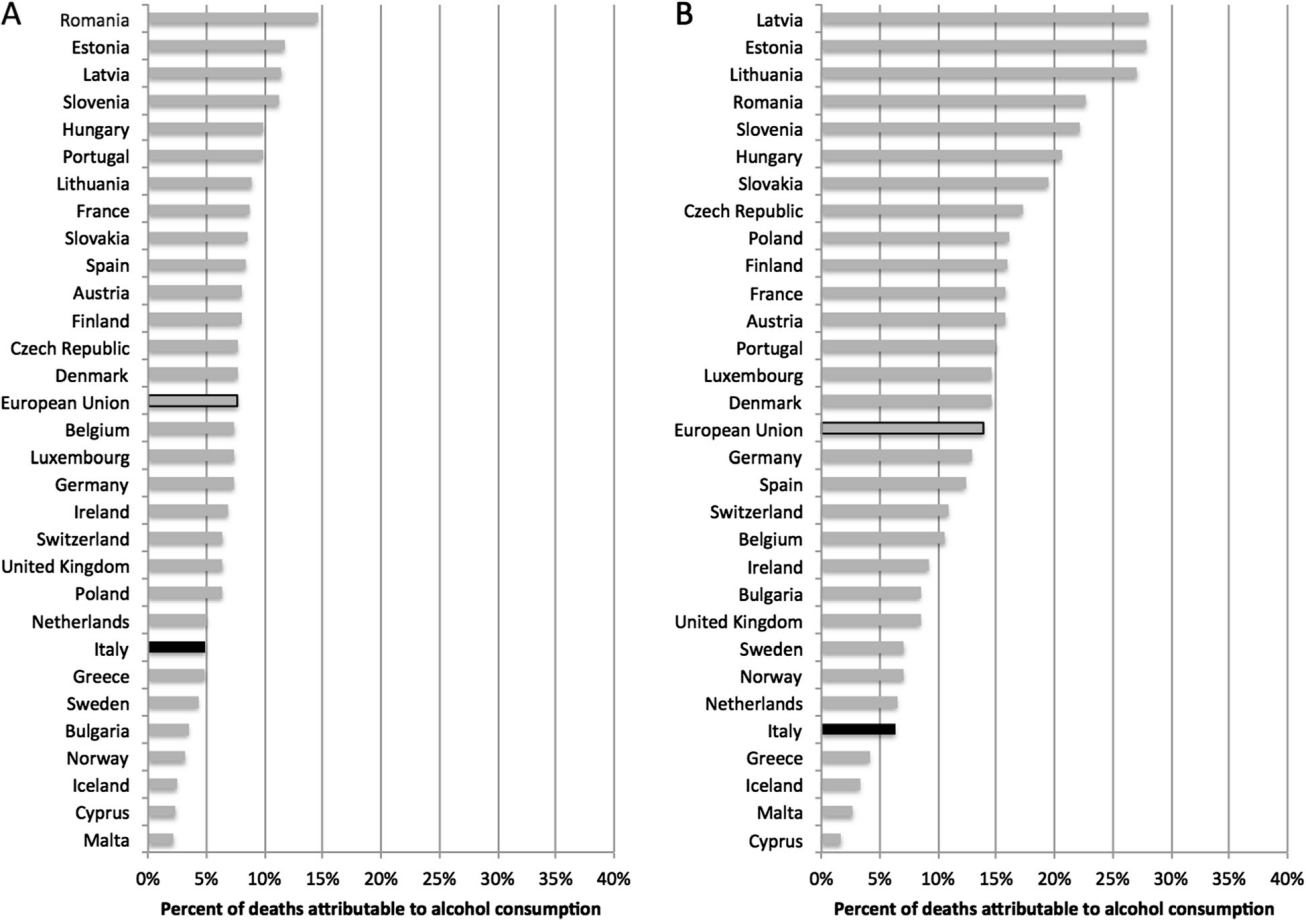
The percentage of deaths attributable to alcohol consumption by country for: A) women, and B) men.

In Italy 5,320 deaths (1,530 female deaths; 3,790 male deaths) of people 15 to 64 years of age were attributable to alcohol in 2004. On average, 7.6 deaths of women and 19.0 deaths of men, per 100,000 people (15 to 64 years of age), were estimated to be attributable to alcohol consumption in 2004, placing Italy 24th out of the 30 countries analyzed (the EU countries plus Iceland, Norway and Switzerland). Alcohol consumption was responsible for 4.9% of all deaths of women and 6.3% of all deaths of men, placing Italy 23rd and 26th, respectively out of the above-mentioned 30 analyzed countries (see Figure 2). Thus, even though the toll of drinking on mortality is relatively low by EU standards, approximately 1 in every 20 women and 1 in every 16 men were estimated to have died prematurely due to alcohol-attributable causes (Figure 2).

In Italy 118,100 DALYs (28,000 female DALYs; 90,100 male DALYs) for people 15 to 64 years of age were attributable to alcohol in 2004. In Italy, 146 DALYs for women and 460 DALYs for men, per 100,000 people (for people 15 to 64 years of age) were estimated to be attributable to alcohol in 2004, placing Italy 28th and 30th for women and men respectively among the examined European countries. The percentage of the total health burden attributable to alcohol as measured in DALYs was 1.6% for women (rank: 28th) and 4.5% for men (rank: 30th) in 2004. Again, while the relative level of burden of disease is low compared to other European countries, the absolute level is relatively high.

**Figure 3:**
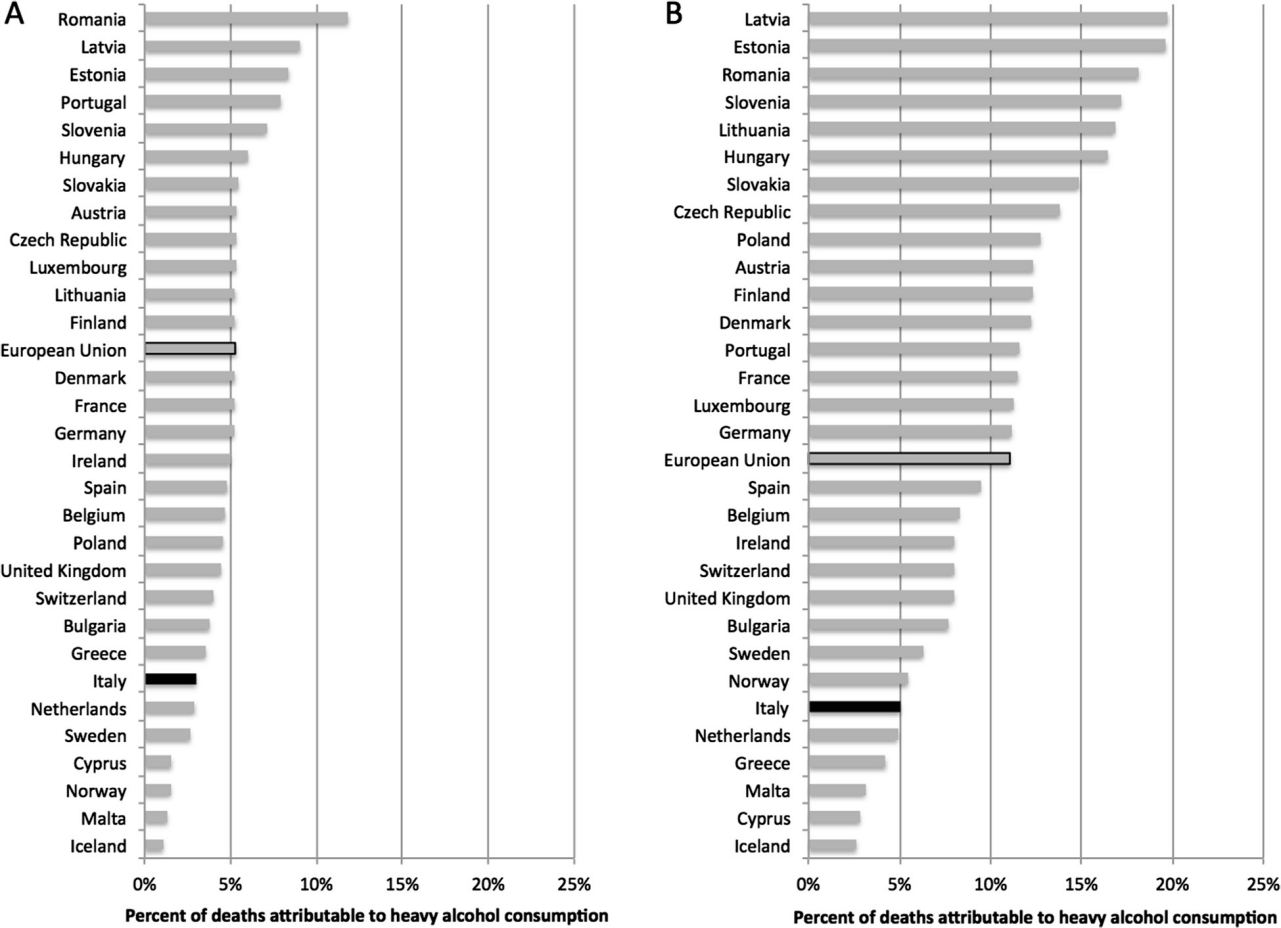
The percentage of deaths attributable to heavy alcohol consumption by country for: A) women, and B) men.

Figure 3 outlines the percentage of all deaths that are attributable to heavy alcohol consumption in Italy in 2004 for men and women. 4.4% (3.0% for women and 5.1% for men) of all deaths were attributable to heavy drinking for people 15 to 64 years of age. Thus, heavy drinking was responsible for 74.5% of the alcohol-attributable deaths (61.3% for women and 79.8% for men).

In Italy 390 female deaths and 1043 male deaths for people 15 to 64 years of age were attributable to AD in 2004. This corresponds to 1.6% (1.3% for females; 1.7% for males) of all deaths for people 15 to 64 years of age being attributable to AD in Italy. Of the total number of alcohol-attributable deaths, 26.9% (25.5% for females; 27.5% for males) of these deaths were attributable to AD.

### Effects of potential treatment interventions

Table 1 outlines the results of simulating the treatment of 40% of people with AD under varying assumptions of AD prevalence and the type of treatment. We observed that the most effective intervention in Italy (if coverage rates were increased to 40%) was pharmacological treatment. If 40% of people with AD in Italy underwent pharmacological treatment in 2004, 3.3% of female alcohol-attributable deaths and 7.6% of male alcohol-attributable deaths would not have occurred. The second most effective treatment was BI, with 2.9% of female alcohol-attributable deaths and 7.4% of male alcohol-attributable deaths not occurring if 40% of people with AD in Italy were treated in that manner.

**Table 1:** The number and percentage of alcohol-attributable deaths in Italy that would not have occurred in 2004 if the treatment coverage for alcohol dependence was 40%

		**Pharmacological treatment**		**Motivational interviewing/cognitive behavioral therapy 1**		**Motivational interviewing/cognitive behavioral therapy 2**		**Brief intervention - hospital 1**		**Brief intervention - hospital 2**	
	**AD estimate**	**Women**	**Men**	**Women**	**Men**	**Women**	**Men**	**Women**	**Men**	**Women**	**Men**
Deaths avoided	Italy (ESMeD study)	50	287	34	130	40	153	32	126	44	279
	Italy [5]	42	131	28	63	29	74	26	62	37	133
	Southern Europe (ESMeD study)	70	523	50	239	59	280	43	232	65	511
	EU (ESMeD study)	121	829	84	390	101	459	81	383	109	840
Percentage of all alcohol-attributable deaths avoided	Italy (ESMeD study)	3.3%	7.6%	2.2%	3.4%	2.6%	4.0%	2.1%	3.3%	2.9%	7.4%
	Italy [5]	2.7%	3.5%	1.8%	1.7%	1.9%	2.0%	1.7%	1.6%	2.4%	3.5%
	Southern Europe (ESMeD study)	4.6%	13.8%	3.3%	6.3%	3.9%	7.4%	2.8%	6.1%	4.2%	13.5%
	EU (ESMeD study)	7.9%	21.9%	5.5%	10.3%	6.6%	12.1%	5.3%	10.1%	7.1%	22.0%

For our sensitivity analysis, we observed that pharmacological treatment was the most effective intervention for both men and women (at a coverage rate of 40%) regardless of the estimated prevalence of AD. We observed that the percentage of alcohol-attributable deaths that would not have occurred if 40% of people with AD were treated with pharmacological treatment would be 2.7% for women and 3.5% for men if we used the AD prevalence estimates from the Scafato and colleagues study, 4.6% for women and 13.8% for men if we used the AD prevalence estimates for Southern Europe, and 7.9% for women and 21.9% for men if we used the AD prevalence estimates for the EU. In general, we observed that the greater the estimated prevalence of AD, the greater the number of deaths that would not have occurred if coverage of AD treatment was at a level of 40%. This relationship was linear and was observed for all five interventions.

## Discussion

In 2004 alcohol consumption in Italy was responsible for approximately 5,320 deaths (1,530 female deaths; 3,790 male deaths) or 5.9% of all deaths (4.9% of all female deaths; 6.3% of all male deaths). Although low when compared to the rest of the EU, given the magnitude of the alcohol-attributable burden of disease in Italy, it is imperative to assess the effectiveness of policies and interventions that can reduce this burden.

AD is responsible for 26.9% of the alcohol-attributable burden of disease in Italy; however, the current AD treatment coverage rate for Italy is low, with less than 10% of people with AD being treated [5]. Accordingly, one area for improvement would be to increase treatment coverage rates for individuals with AD [2,28]. In particular, if coverage of pharmacological AD treatment (observed to be the most effective form of treatment) were to increase to 40% of all people with AD, we estimated the resulting prevention of 3.3% (50 deaths) of all female and 7.6% (287 deaths) of all male alcohol-attributable deaths in 2004 in Italy. The introduction of BI in hospitals would result in the prevention of a substantial number of deaths, as people in hospitals have a higher risk of mortality, and reducing alcohol consumption within this population group would have a greater effect in reducing mortality than if this form of intervention was administered to the general population; according to the latest Cochrane analyses, the mortality rate was reduced by 40% within 1 year of the BI treatment intervention [27].

Psychotherapy, sometimes combined with pharmacotherapy, is the most common type of AD therapy in Italy. The most commonly used psychotherapeutic procedures (from most frequent to less frequent) are MI, self-help group facilitation, BI, CBT, and psychodynamic interventions (based on unpublished study findings). Based on our findings, increasing the proportion of people with AD who undergo pharmacological treatment could help to reduce the alcohol-attributable burden of disease in Italy. Furthermore, it is notable that the treatment coverage rate for depression (another neuropsychiatric disorder) has been increased to 40% in developing nations and, thus, a 40% treatment coverage rate for AD may be feasible [34].

It should be noted that the feasibility costs and time to increase the coverage rate of AD treatment to 40% are not assessed in this article. We also did not compare the cost effectiveness of increasing the AD coverage rate to 40% with general population interventions that aim to reduce population consumption, as cost estimates for increasing AD therapy are not available.

### Limitations

Our analysis has certain limitations. Information concerning the cause of death has been shown to contain inaccuracies [35], with recent studies confirming considerable degrees of error in such information [36,37]. To minimize the error from the unreliable nature of mortality data, especially for people older than 64 years of age, we excluded individuals who were 65 years or older when performing our analyses.

Exposure estimates for drinking status and binge drinking patterns used in our analysis were measured in 2009, whereas deaths, PYLL, YLD and DALYs were measured in 2004. The short length of time between 2004 and 2009 should not greatly affect the alcohol-attributable mortality estimates for Italy, as alcohol consumption in Italy has only decreased slightly during this time period [7]; however, this slight decrease in consumption may lead to an underestimation of the number of alcohol-attributable deaths and deaths attributable to AD in 2004. The estimates of alcohol consumption used in our analysis were cross-sectional (i.e., measured concurrently with deaths). Since long-term patterns of alcohol consumption impact the risk of some chronic diseases, such as cancer, there will be resulting inaccuracies in the number of some chronic disease deaths [38]. Furthermore, our analysis did not include aspects of harms to others (such as motor vehicle accidents, and assaults), which recently have been shown to constitute a large proportion of the burden of injury attributable to alcohol [39].

Additionally, the mortality data, AD prevalence data, and AD treatment coverage data used in our analysis was not available for regions within Italy. Thus, our analysis was limited as we could not estimate the variability in the burden of alcohol consumption, heavy alcohol consumption and AD; and the potential number of deaths that would have been avoided if the treatment coverage was increased to 40% of all people with AD in 2004 for regions within Italy as alcohol consumption in Italy has been shown to vary, the effect of increasing treatment coverage for AD should vary by region in Italy [40]. Additionally, the data on alcohol dependence RRs were not estimated by age and, thus, an analysis of increasing AD treatment coverage rates by age was not possible.

The mortality data used in our analysis did not have a measure of uncertainty and, accordingly, our analysis was unable to provide 95% confidence intervals for the number of deaths that could have been avoided in 2004 if the treatment coverage of AD was increased to 40%. Additionally, the measures used in this analysis to estimate the number of deaths that would have been avoided in 2004 if the treatment coverage rate of people with AD increased to 40% have random error associated with them, and, thus, these estimates do have some uncertainty that is not reported in this paper.

The RRs used in this study for general causes also have inaccuracies. First, observational studies that measured the alcohol RR are susceptible to the underreporting of alcohol consumption [41]. To account for this, we modelled alcohol consumption using 80% of adult *per capita* consumption to account for alcohol not consumed and the underreporting of alcohol consumption in observational studies [12,13]. Second, most analyses on the risk of alcohol-related conditions do not distinguish between incidence-based alcohol RRs and mortality- based alcohol RRs; the RRs for mortality and morbidity from alcohol-related conditions differ in some cases which may lead to our results being underestimates (as morbidity alcohol RRs are often lower than mortality alcohol RRs [42]). Third, the RR functions used in our analysis are adjusted; however, the AAF formulas were designed for unadjusted RR functions. When alcohol RR functions are adjusted for the usual confounders, the adjusted RRs are not noticeably different when compared to unadjusted RRs, and, thus, the use of adjusted RRs will not noticeably affect estimates of alcohol-attributable mortality (see [43] for an in-depth discussion of using adjusted alcohol RRs to calculate alcohol-attributable mortality).

In Italy long-term γ-hydroxybutyric acid (ghb), naltrexone, and disulfiram are used as main treatments for alcohol dependence [4]; however, there are unfortunately not enough trials of these medications for a comprehensive meta-analysis and, thus, we only modelled the effects of acamprosate and opioid antagonists therapy. We expect that the conclusions of this study would not change tremendously with other medications as examples, if based on a large number of trials. In addition, the current model of acamprosate and opioid antagonists therapy allows for comparisons with other European countries, such as Spain [44].

## Conclusions

Alcohol consumption is a major risk factor for mortality in Italy. More substantial alcohol policies and improved coverage of pharmacological treatment for people with AD could reduce alcohol consumption and the resulting burden of disease. Effective improvement of the coverage rates of AD treatments in Italy would result in the prevention of a large number of alcohol-attributable deaths since AD treatment coverage rates are estimated to be very low. Thus, implementing measures aimed at reducing alcohol consumption and increasing AD treatment coverage rates should be considered by policy makers in Italy in order to reduce the country’s large alcohol-attributable burden of disease.

## Abbreviations

AA: Alcoholic anonymous; AAF: Alcohol-attributable fraction; AD: Alcohol dependence; BI: Brief interventions; CAT: Club of treated alcoholics; DALYs: Disability adjusted years of life; DSM IV: Diagnostic and statistical manual of mental disorders (version IV); EU: European union; GBD: Global burden of disease; GENACIS: GENder alcohol and culture international study; l: Litres; MI/CBT: Motivational interviewing/cognitive behavioral therapy; PYLL: Potential years of life lost; RR: Relative risk; YLD: Years lived with disability.

## Competing interests

None declared. Dr. Rehm has received an unrestricted grant from the Lundbeck to study alcohol related harms in the European Union.

## Authors’ contributions

KS, JR, and AA conceptualized the overall article. KS, JR, GG and AA contributed to the methodology. KS, JR, GG, MR and AA identified sources for risk relations and exposure, and contributed to the writing of the manuscript. KS and GG performed all statistical analyses. All authors read and approved the final manuscript.

## Supplementary Material

Additional file 1 Categories of alcohol-related diseases and sources used for determining Alcohol-Attributable Fractions (AAFs). Click here for file

Additional file 2 Overview of the assumptions used when modelling alcohol dependence interventions. Click here for file

Additional file 3 Country profile for Italy. Click here for file
