# Supplementary material for: Alcohol consumption, alcohol dependence, and related mortality in Italy in 2004: effects of treatment-based interventions on alcohol dependence
Source: Subst Abuse Treat Prev Policy. 2013 Jun 13;8:21. doi: 10.1186/1747-597X-8-21 (PMC3686709; doi:10.1186/1747-597X-8-21)
Supplement: Additional file 1 — Categories of alcohol-related diseases and sources used for determining Alcohol-Attributable Fractions (AAFs). [file 1747-597X-8-21-S1.docx]

# Additional file 2. Categories of alcohol-related disease and sources used for determining Alcohol-Attributable Fractions (AAFs)

| **Condition** | | **ICD 10 Code** | **Source for AAF** |
| --- | --- | --- | --- |
| **Infectious and parasitic diseases** | |  |  |
|  | Tuberculosis | A15-A19 | ([Lönnroth et al., 2008](#_ENREF_7)); for causal relationship see: ([Rehm et al., 2009](#_ENREF_11)) |
| **Human immunodeficiency virus/ Acquired immune deficiency syndrome** | | B20-B24 | ([Gmel et al., 2011](#_ENREF_5)) |
| **Malignant neoplasm's** | |  |  |
|  | Mouth and oropharynx cancers | C00-C14 | ([Baan et al., 2007](#_ENREF_1), [Cancer, 2010](#_ENREF_3)) (based on Relative Risks from ([Corrao et al., 2004](#_ENREF_4))) |
|  | Esophageal cancer | C15 | ([Baan et al., 2007](#_ENREF_1), [Cancer, 2010](#_ENREF_3)) (based on Relative Risks from ([Corrao et al., 2004](#_ENREF_4))) |
|  | Liver cancer | C22 | ([Baan et al., 2007](#_ENREF_1), [Cancer, 2010](#_ENREF_3)) (based on Relative Risks from ([Corrao et al., 2004](#_ENREF_4))) |
|  | Laryngeal cancer | C32 | ([Baan et al., 2007](#_ENREF_1), [Cancer, 2010](#_ENREF_3)) (based on Relative Risks from ([Corrao et al., 2004](#_ENREF_4))) |
|  | Breast cancer | C50 | ([Baan et al., 2007](#_ENREF_1), [Cancer, 2010](#_ENREF_3)) (based on Relative Risks from ([Corrao et al., 2004](#_ENREF_4))) |
|  | Colon cancer | C18 | ([Baan et al., 2007](#_ENREF_1), [Cancer, 2010](#_ENREF_3)) (based on Relative Risks from ([Corrao et al., 2004](#_ENREF_4))) |
|  | Rectal cancer | C20 | ([Baan et al., 2007](#_ENREF_1), [Cancer, 2010](#_ENREF_3)) (based on Relative Risks from ([Corrao et al., 2004](#_ENREF_4))) |
| **Diabetes** | |  |  |
|  | Diabetes mellitus | E10-E14 | ([Baliunas et al., 2009](#_ENREF_2)) |
| **Neuro-psychiatric conditions** | |  |  |
|  | Alcoholic psychoses  (part of AUD) | F10.0, F10.3-F10.9 | 100% AAF per definition |
|  | Alcohol abuse (part of AUD) | F10.1 | 100% AAF per definition |
|  | Alcohol dependence (part of AUD) | F10.2 | 100% AAF per definition |
|  | Epilepsy | G40-G41 | ([Samokhvalov et al., 2010a](#_ENREF_14)) |
| **Cardiovascular disease** | |  |  |
|  | Hypertensive disease | I10-I15 | ([Taylor et al., 2009](#_ENREF_17)) |
|  | Ischemic heart disease | I20-I25 | ([Roerecke and Rehm, 2012](#_ENREF_13)), for volume, ([Roerecke and Rehm, 2010](#_ENREF_12)) for pattern |
|  | Cardiac arrhythmias | I47-I49 | ([Samokhvalov et al., 2010b](#_ENREF_15)) |
|  | Haemorrhagic and other non-ischaemic stroke | I60–I62, I69.0, I69.1, I69.2 | ([Patra et al., 2010](#_ENREF_9)) |
|  | Ischaemic stroke | I63–I67, I69.3 | ([Patra et al., 2010](#_ENREF_9)) |
| **Digestive diseases** | |  |  |
|  | Cirrhosis of the liver | K70, K74 | ([Rehm et al., 2010](#_ENREF_10)) |
|  | Acute and chronic pancreatitis | K85, K86.1 | ([Irving et al., 2009](#_ENREF_6)) |
| **Respiratory infections** | |  |  |
|  | Pneumonia | J10.0, J11.0, J12-J15, J18 | ([Samokhvalov et al., 2010c](#_ENREF_16)) |
| **Conditions arising during the prenatal period** | |  |  |
|  | Low birth weight: as defined by the global burden of disease | P05-P07 | ([Patra et al., 2011](#_ENREF_8)) |
| **Unintentional injuries** | |  |  |
|  | Motor vehicle accidents | § | ([Taylor et al., 2010](#_ENREF_18)) for Relative Risk, methodology adopted from ([Taylor et al., 2011](#_ENREF_19)) |
|  | Poisonings | X40-X49 | ([Taylor et al., 2010](#_ENREF_18)) for Relative Risk, methodology adopted from ([Taylor et al., 2011](#_ENREF_19)) |
|  | Falls | W00-W19 | ([Taylor et al., 2010](#_ENREF_18)) for Relative Risk, methodology adopted from ([Taylor et al., 2011](#_ENREF_19)) |
|  | Fires | X00-X09 | ([Taylor et al., 2010](#_ENREF_18)) for Relative Risk, methodology adopted from ([Taylor et al., 2011](#_ENREF_19)) |
|  | Drowning | W65-W74 | ([Taylor et al., 2010](#_ENREF_18)) Relative Risk, methodology adopted from ([Taylor et al., 2011](#_ENREF_19)) |
|  | Other Unintentional injuries | †Rest of V-series and W20-W64, W 75-W99, X10-X39, X50-X59, Y40-Y86, Y88, and Y89 | ([Taylor et al., 2010](#_ENREF_18)) for Relative Risk, methodology adopted from ([Taylor et al., 2011](#_ENREF_19)) |
| **Intentional injuries** | |  | ([Taylor et al., 2010](#_ENREF_18)) for Relative Risk, methodology adopted from ([Taylor et al., 2011](#_ENREF_19)) |
|  | Self-inflicted injuries | X60-X84 and Y87.0 | ([Taylor et al., 2010](#_ENREF_18)) for Relative Risk, methodology adopted from ([Taylor et al., 2011](#_ENREF_19)) |
|  | Homicide | X85-Y09, Y87.1 | ([Taylor et al., 2010](#_ENREF_18)) for Relative Risk, methodology adopted from ([Taylor et al., 2011](#_ENREF_19)) |
|  | Other intentional injuries |  | ([Taylor et al., 2010](#_ENREF_18)) for Relative Risk, methodology adopted from ([Taylor et al., 2011](#_ENREF_19)) |
| § V021–V029, V031–V039, V041–V049, V092, V093, V123–V129, V133–V139, V143–V149, V194–V196, V203–V209, V213–V219, V223–V229, V233–V239, V243–V249,V253–V259, V263–V269, V273– V279, V283–V289, V294–V299, V304–V309, V314–V319, V324–V329, V334–V339, V344–V349, V354–V359, V364–V369, V374–V379, V384–V389, V394–V399, V404–V409, V414–V419, V424–V429, V434–V439, V444–V449, V454–V459, V464– V469, V474–V479, V484–V489, V494–V499, V504–V509, V514–V519, V524–V529, V534–V539, V544–V549, V554–V559, V564–V569, V574–V579, V584–V589, V594–V599, V604–V609, V614–V619, V624–V629, V634–V639, V644–V649, V654– V659, V664–V669, V674–V679, V684–V689, V694–V699, V704–V709, V714–V719, V724–V729, V734–V739, V744–V749, V754–V759, V764–V769, V774–V779, V784–V789, V794–V799, V803–V805, V811, V821, V830–V833, V840–V843, V850– V853, V860–V863, V870–V878, V892.  †Rest of V = V-series MINUS §. | | | |

References

Allamani, A, & Prina, F. (2007). Why the decrease in consumption of alcoholic beverages in Italy between the 1970's and 2000's? shedding light on an Italian mystery. *Contemp Drug Probl* 34: 187-198

American Psychiatric Association. (2000). Diagnostic and statistical manual of mental disorders, text revision. Washington, D.C.: American Psychiatric Association.

Anderson, P, & Baumberg, B. (2006). Alcohol in Europe. A Public Health Perspective. United Kingdom: Institute of Alcohol Studies for the European Commission London.

Baan, R, Straif, K, Grosse, Y, Secretan, B, El Ghissassi, F, Bouvard, V, Alteri, A, & Cogliano, V, On behalf of the WHO International Agency for Research on Cancer monograph working group. (2007). Carcinogenicity of alcoholic beverages. *Lancet Oncol* 8: 292-293

Baliunas, D, Taylor, B, Irving, H, Roerecke, M, Patra, J, Mohapatra, S, & Rehm, J. (2009). Alcohol as a risk factor for type 2 diabetes - A systematic review and meta-analysis. *Diabetes Care* 32: 2123-2132

Corrao, G, Bagnardi, V, Zambon, A, & La Vecchia, C. (2004). A meta-analysis of alcohol consumption and the risk of 15 diseases. *Prev Med* 38: 613-619

Cuijpers, P, Riper, H, & Lemmers, L. (2004). The effects on mortality of brief interventions for problem drinking: a meta-analysis. *Addiction* 99: 839-845

de Girolamo, G, Polidori, G, Morosini, P, Scarpino, V, Reda, V, Serra, G, Mazzi, F, Alonso, J, Vilagut, G, Visona, G, Falsirollo, F, Rossi, A, & Warner, R. (2006). Prevalence of common mental disorders in Italy: results from the European Study of the Epidemiology of Mental Disorders (ESEMeD). *Soc Psychiatry Psychiatr Epidemiol* 41: 853-861

De Lorenze, GN, Weisner, C, Tsai, AL, Satre, DD, & Quesenberry, CPJ. (2011). Excess mortality among HIV-infected patients diagnosed with substance use dependence or abuse receiving care in a fully integrated medical care program. *Alcohol Clin Exp Res* 35: 203-210

European Commission. (2010). EU citizens' attitudes towards alcohol. Special Eurobarometer 331. Brussels, Belgium: TNS.

Gmel, G, Shield, K, & Rehm, J. (2011). Developing a methodology to derive alcohol-attributable fractions for HIV/AIDS mortality based on alcohol's impact on adherence to antiretroviral medication. *Popul Health Metr* 9: 5

Grant, BF, Compton, WM, Crowley, TJ, Hasin, DS, Helzer, JE, Li, TK, Rounsaville, BJ, Volkow, ND, & Woody, GE. (2007). Errors in assessing DSM-IV substance use disorders. *Arch Gen Psychiatry* 64: 379-380

Gual, A, & Colom, J. (1997). Why has alcohol consumption declined in countries of southern Europe? *Addiction* 92: S21-S31

Harris, EC, & Barraclough, B. (1998). Excess mortality of mental disorder. *Br J Psychiatry* 173: 11-53

International Agency for Research on Cancer. (2010). Alcoholic beverage consumption and ethyl carbamate (urethane). IARC Monograph 96 on the Evaluation of Carcinogenic Risks to Humans. Lyon, France: International Agency for Research on Cancer (IARC).

Irving, HM, Samokhvalov, A, & Rehm, J. (2009). Alcohol as a risk factor for pancreatitis. A systematic review and meta-analysis. *JOP* 10: 387-392

Italian Ministry of Health. (2011). Relazione del Ministro della Salute al Parlamento sugli interventi realizzati ai Sensi della Legge 30.3.2001 N. 125 "Legge Quadro In Materia di Alcol e Problemi Alcolcorrelati" [Report to Parliament from the Italian Ministry of Health about interventions according to law 30.3.2001 number 125 "General Law on Alcohol and Alcohol -related problems" ]. Rome, Italy: Italian Ministry of Health.

Jacobi, F, Wittchen, H, Holting, C, Hofler, M, Pfister, H, Muller, N, & Lieb, R. (2004). Prevalence, co-morbidity and correlates of mental disorders in the general population: results from the German Health Interview and Examination Survey (GHS). *Psychol Med* 34: 1-15

Jacobi, F, Wittchen, H, Holting, C, Sommer, S, & Lieb, R. (2002). Estimating the prevalence of mental and somatic disorders in the community: aims and methods of the German National Health Interview and Examination Survey. *Int J Methods Psychiatr Res* 11: 1-18

Lönnroth, K, Williams, B, Stadlin, S, Jaramillo, E, & Dye, C. (2008). Alcohol use as a risk factor for tuberculosis - a systematic review. *BMC Public Health* 8: 289

Lopez, AD, Mathers, CD, Ezzati, M, Jamison, DT, Murray, CJL. (2006). Measuring the global burden of disease and risk factors, 1990-2001., In Lopez AD et al. (eds), *Global burden of disease and risk factors.*, pp. 1-13. Washington, DC:World Bank.

Magill, M, & Ray, LA. (2009). Cognitive-behavioral treatment with adult alcohol and illicit drug users: ameta-analysis of randomized controlled trials. *J Stud Alcohol Drugs* 70: 516-527

McQueen, J, Howe, TE, Allan, L, Mains, D, & Hardy, V. (2011). Brief interventions for heavy alcohol users admitted to general hospital wards. *Cochrane Database Syst Rev* 8: CD005191

O'Brien, JMJ, Lu, B, Ali, NA, Martin, GS, Aberegg, SK, Marsh, CB, Lemeshow, S, & Douglas, IS. (2007). Alcohol dependence is independently associated with sepsis, septic shock, and hospital mortality among adult intensive care unit patients. *Critical Care Medicine* 35: 345-350

Osservatorio Permanente sui Giovani e l'Alcool. (2007). Italians and Alcohol. Consumption, Trend and Attitudes. 5^th^ Doxa National Survey. Litos, Roma: Osservatorio Permanente sui Giovani e l'Alcool.

Patra, J, Taylor, B, Irving, H, Roerecke, M, Baliunas, D, Mohapatra, S, & Rehm, J. (2010). Alcohol consumption and the risk of morbidity and mortality from different stroke types - a systematic review and meta-analysis. *BMC Public Health* 10: 258

Patra, J, Bakker, R, Irving, H, Jaddoe, VWV, Malini, S, & Rehm, J. (2011). Dose-response relationship between alcohol consumption before and during pregnancy and the risks of low birthweight, preterm birth and small for gestational age (SGA)-a systematic review and meta-analyses. *BJOG: International Journal of Obstetrics and Gynaecology* 118: 1411-1421 10.1111/j.1471-0528.2011.03050.x.

Patussi, V, Tumino, E, & Poldrugo, F. (1996). The development of the Alcoholic Treatment Club system in Italy: fifteen years of experience. *Contemp Drug Probl* 23: 29-42

Project MATCH Research Group. (1997). Matching alcoholism treatment to client heterogeneity: Project MATCH posttreatment drinking outcomes. *J Stud Alcohol* 58: 7-30

Rehm, J, Kehoe, T, Gmel, G, Stinson, F, Grant, B, & Gmel, G. (2010). Statistical modeling of volume of alcohol exposure for epidemiological studies of population health: the example of the US. *Popul Health Metr* 8: 3

Rehm, J, Mathers, C, Popova, S, Thavorncharoensap, M, Teerawattananon, Y, & Patra, J. (2009). Global burden of disease and injury and economic cost attributable to alcohol use and alcohol use disorders. *Lancet* 373: 2223-2233

Rehm, J, Rehn, N, Room, R, Monteiro, M, Gmel, G, Jernigan, D, & Frick, U. (2003). The global distribution of average volume of alcohol consumption and patterns of drinking. *Eur Addict Res* 9: 147-156

Rehm, J, Room, R, Monteiro, M, Gmel, G, Graham, K, Rehn, N, Sempos, CT, Frick, U, & Jernigan, D. (2004). Alcohol Use, In Ezzati M et al. (eds), *Comparative quantification of health risks: global and regional burden of disease attributable to selected major risk factors*, vol. 1, pp. 959-1109. Geneva, Switzerland:World Health Organization.

Rehm, J, Shield, K, Rehm, M, Gmel, Gj, & Frick, U. (2012). Alcohol consumption, alcohol dependence, and attributable burden of disease: potential gains from effective interventions for alcohol dependence. Toronto, ON: Centre for Addiction and Mental Health.

Roerecke, M, & Rehm, J. (2010). Irregular heavy drinking occasions and risk of ischemic heart disease: a systematic review and meta-analysis. *Am J Epidemiol* 171: 633-644

Roerecke, M, & Rehm, J. (2012). The cardioprotective association of average alcohol consumption and ischaemic heart disease: a systematic review and meta-analysis. *Addiction* [Epub ahead of print]:

Room, R, Babor, T, & Rehm, J. (2005). Alcohol and public health: a review. *Lancet* 365: 519-530

Rösner, S, Hackl-Herrwerth, A, Leucht, S, Lehert, P, Vecchi, S, & Soyka, M. (2010a). Acamprosate for alcohol dependence. *Cochrane Database Syst Rev* 9: CD004332

Rösner, S, Hackl-Herrwerth, A, Leucht, S, Vecchi, S, Srisurapanont, M, & Soyka, M. (2010b). Opioid antagonists for alcohol dependence. *Cochrane Database Syst Rev* 12: CD001867

Samokhvalov, AV, Irving, H, Mohapatra, S, & Rehm, J. (2010a). Alcohol consumption, unprovoked seizures and epilepsy: a systematic review and meta-analysis. *Epilepsia* 51: 1177-1184 Doi:10.1111/j.1528-1167.2009.02426.x.

Samokhvalov, AV, Irving, HM, & Rehm, J. (2010b). Alcohol as a risk factor for atrial fibrillation: a systematic review and meta-analysis. *Eur J Cardiovasc Prev Rehabil* 17: 706-712

Samokhvalov, AV, Irving, HM, & Rehm, J. (2010c). Alcohol consumption as a risk factor for pneumonia: systematic review and meta-analysis. *Epidemiol Infect* 138: 1789-1795

Scafato, E, Allamani, A, Patussi, V, Codenotti, T, Marcomini, F, Struzzo, P, the Italian WHO Phase IV EIBI Working Group. (2005). Italy, In Heather N (ed), *WHO Collaborative project on identification and managment of alcohol-related problems in primary health care - Report on Phase IV*, pp. 131-144. Geneva, Switzerland:World Health Organization - Department of Mental Health and Substance Abuse.

Scafato, E, Ghirini, S, Galluzzo, L, Farchi, G, & Gandin, C. (2009). Rapporto su raccolta e analisi centralizzata dei flussi informativi e dati per il monitoraggio dell'impatto dell'uso e abuso dell'alcol sulla salute in Italia. [Report on the centralised collection and analysis of information and data about monitoring the impact of alcohol use and abuse on health in Italy] Rome, Italy: Osservatorio Nazionale Alcol CNESPS, Istituto Superiore di Sanità.

Smedslund, G, Berg, RC, Hammerstrom, KT, Steiro, A, Leiknes, KA, Dahl, HM, & Karlsen, K. (2011). Motivational interviewing for substance abuse. *Cochrane Database Syst Rev* 5: CD008063

Sulkunen, P. (1989). Drinking in France 1965-1979. An analysis of household consumption data. *Br J Addict* 84: 61-72

Taylor, B, Irving, HM, Baliunas, D, Roerecke, M, Patra, J, Mohapatra, S, & Rehm, J. (2009). Alcohol and hypertension: gender differences in dose-response relationships determined through systematic review and meta-analysis. *Addiction* 104: 1981-1990

Taylor, B, Irving, HM, Kanteres, F, Room, R, Borges, G, Cherpitel, C, Greenfield, T, & Rehm, J. (2010). The more you drink, the harder you fall: a systematic review and meta-analysis of how acute alcohol consumption and injury or collision risk increase together. *Drug Alcohol Depend* 110: 108-116 DOI: 10.1016/j.drugalcdep.2010.02.011.

Taylor, B, Shield, K, & Rehm, J. (2011). Combining best evidence: a novel method to calculate the alcohol-attributable fraction and its variance for injury mortality. *BMC Public Health* 11: 265

Voller, F. (2007). Trends in alcoholic beverage consumption in Italy. *Contemp Drug Probl* 34: 199-226

World Health Organization. (2011). Global status report on alcohol and health. Geneva, Switzerland: World Health Organization.

Reference List

Baan, R., Straif, K., Grosse, Y., Secretan, B., El Ghissassi, F., Bouvard, V., Alteri, A., Cogliano, V. & Group, O. B. O. T. W. I. a. F. R. O. C. M. W. 2007. Carcinogenicity of alcoholic beverages. *Lancet Oncology,* 8**,** 292-293.

Baliunas, D., Taylor, B., Irving, H., Roerecke, M., Patra, J., Mohapatra, S. & Rehm, J. 2009. Alcohol as a risk factor for type 2 diabetes - A systematic review and meta-analysis. *Diabetes Care,* 32**,** 2123-2132.

Cancer, I. a. F. R. O. 2010. IARC Monograph 96 on the Evaluation of Carcinogenic Risks to Humans. Alcoholic beverage consumption and ethyl carbamate (urethane). Lyon, France.

Corrao, G., Bagnardi, V., Zambon, A. & La Vecchia, C. 2004. A meta-analysis of alcohol consumption and the risk of 15 diseases. *Preventive Medicine,* 38**,** 613-619.

Gmel, G., Shield, K. & Rehm, J. 2011. Developing a methodology to derive alcohol-attributable fractions for HIV/AIDS mortality based on alcohol's impact on adherence to antiretroviral medication. *Population Health Metrics,* 9**,** 5.

Irving, H. M., Samokhvalov, A. & Rehm, J. 2009. Alcohol as a risk factor for pancreatitis. A systematic review and meta-analysis. *Journal of the Pancreas,* 10**,** 387-392.

Lönnroth, K., Williams, B., Stadlin, S., Jaramillo, E. & Dye, C. 2008. Alcohol use as a risk factor for tuberculosis - a systematic review. *BMC Public Health,* 8**,** 289.

Patra, J., Bakker, R., Irving, H., Jaddoe, V. W. V., Malini, S. & Rehm, J. 2011. Dose-response relationship between alcohol consumption before and during pregnancy and the risks of low birthweight, preterm birth and small for gestational age (SGA)-a systematic review and meta-analyses. *BJOG: International Journal of Obstetrics and Gynaecology,* 118**,** 1411-1421.

Patra, J., Taylor, B., Irving, H., Roerecke, M., Baliunas, D., Mohapatra, S. & Rehm, J. 2010. Alcohol consumption and the risk of morbidity and mortality from different stroke types - a systematic review and meta-analysis. *BMC Public Health,* 10**,** 258.

Rehm, J., Kehoe, T., Gmel, G., Stinson, F., Grant, B. & Gmel, G. 2010. Statistical modeling of volume of alcohol exposure for epidemiological studies of population health: the example of the US. *Population Health Metrics,* 8**,** 3.

Rehm, J., Mathers, C., Popova, S., Thavorncharoensap, M., Teerawattananon, Y. & Patra, J. 2009. Global burden of disease and injury and economic cost attributable to alcohol use and alcohol use disorders. *Lancet,* 373**,** 2223-2233.

Roerecke, M. & Rehm, J. 2010. Irregular heavy drinking occasions and risk of ischemic heart disease: a systematic review and meta-analysis. *American Journal of Epidemiology,* 171**,** 633-644.

Roerecke, M. & Rehm, J. 2012. The cardioprotective association of average alcohol consumption and ischaemic heart disease: a systematic review and meta-analysis. *Addiction,* 107**,** 1246-1260.

Samokhvalov, A. V., Irving, H., Mohapatra, S. & Rehm, J. 2010a. Alcohol consumption, unprovoked seizures and epilepsy: a systematic review and meta-analysis. *Epilepsia,* 51**,** 1177-1184.

Samokhvalov, A. V., Irving, H. M. & Rehm, J. 2010b. Alcohol as a risk factor for atrial fibrillation: a systematic review and meta-analysis. *European Journal of Cardiovascular Prevention & Rehabilitation,* 17**,** 706-712.

Samokhvalov, A. V., Irving, H. M. & Rehm, J. 2010c. Alcohol consumption as a risk factor for pneumonia: systematic review and meta-analysis. *Epidemiology and Infection,* 138**,** 1789-1795.

Taylor, B., Irving, H. M., Baliunas, D., Roerecke, M., Patra, J., Mohapatra, S. & Rehm, J. 2009. Alcohol and hypertension: gender differences in dose-response relationships determined through systematic review and meta-analysis. *Addiction,* 104**,** 1981-1990.

Taylor, B., Irving, H. M., Kanteres, F., Room, R., Borges, G., Cherpitel, C., Greenfield, T. & Rehm, J. 2010. The more you drink, the harder you fall: a systematic review and meta-analysis of how acute alcohol consumption and injury or collision risk increase together. *Drug and Alcohol Dependence,* 110**,** 108-116.

Taylor, B., Shield, K. & Rehm, J. 2011. Combining best evidence: a novel method to calculate the alcohol-attributable fraction and its variance for injury mortality. *BMC Public Health,* 11**,** 265.
