# Supplementary material for: Alcohol consumption, alcohol dependence, and related mortality in Italy in 2004: effects of treatment-based interventions on alcohol dependence
Source: Subst Abuse Treat Prev Policy. 2013 Jun 13;8:21. doi: 10.1186/1747-597X-8-21 (PMC3686709; doi:10.1186/1747-597X-8-21)
Supplement: Additional file 2 — Overview of the assumptions used when modelling alcohol dependence interventions. [file 1747-597X-8-21-S2.docx]

## Additional file 3. Overview of assumptions used when modelling interventions

| **Interventions** | **Main results (effects assumed to be stable for 1 year)** | **Risk Relations** | **Sources** |
| --- | --- | --- | --- |
| **MI and CBT 1** | For MI an average drop of 15.8 g of pure alcohol per day was assumed (measured against no intervention; 95% CI from -9.6 g to  -21.8 g of pure alcohol). The effect after one year was very small and not significant (average: 1.2 g of pure alcohol reduction per day), the average effect over the year was a 3.2 g reduction of pure alcohol per day (95% CI:  -1.2 g to -5.2 g of pure alcohol per day).  For CBT almost the same effect was found in studies with a no-treatment control as the comparison condition (15.9 g of pure alcohol per day). In addition, Project Match did not find any significant differences ([Group, 1997](#_ENREF_4)) between MI and CBT.  We modelled the results based on a drop of 15.8 g per day over the year. | The usual dose-dependent risk relations between average consumption of alcohol and disease outcomes were used, multiplied by 2 to account for the overall higher mortality risk of people with AD ([Harris and Barraclough, 1998](#_ENREF_5)). For injury, the RR from ([Harris and Barraclough, 1998](#_ENREF_5)) was used for AD and the risks from ([Corrao et al., 2004](#_ENREF_1)) for non-dependent people. | MI: ([Smedslund et al., 2011](#_ENREF_12))  CBT: ([Magill and Ray, 2009](#_ENREF_6)) |
| **MI and CBT 2** | An average drop of 21.8 g of pure alcohol per day was assumed as the upper limit of the CI for MI/CBT (see above). We assumed proportional CIs compared to the first MI/CBT scenario. |  | ([Smedslund et al., 2011](#_ENREF_12)) |
| **BI 1** | An average drop of 13.5 g of pure alcohol per day with a 95% CI from -2.7 to -24.5 of pure alcohol per day. |  | ([McQueen et al., 2011](#_ENREF_7), [Room et al., 2005](#_ENREF_9)) |
| **BI 2** | An average reduction of the RR for mortality by 0.6 (95% CI: 0.40 to 0.91). This scenario represents the “best case” for BI, as hospitalization is linked to mortality, and AD plays an important role in mediating and moderating this premature mortality (e.g. ([O'Brien et al., 2007](#_ENREF_8), [De Lorenze et al., 2011](#_ENREF_3))). However, similar effects were obtained in an meta-analyses on all BIs ([Cuijpers et al., 2004](#_ENREF_2)). |  | ([McQueen et al., 2011](#_ENREF_7)) |
| **Pharmacological therapy (for simulation, the effects of Randomized Controlled Trials of acamprosate and opioid antagonist treatments were combined** | Overall, for 55.0% of the patient population a reduction in drinking by 13% on average; for 18.1% of the patient population there was a reduction in drinking by 50%; and for 26.8% of the population abstinence was the result. |  | Pooled estimates of ([Rösner et al., 2010b](#_ENREF_11), [Rösner et al., 2010a](#_ENREF_10)). For this simulation we are concerned with the differences in consumption between baseline and follow-up in the group receiving medications only. |

**Reference List**

Corrao, G., Bagnardi, V., Zambon, A., & Vecchia, C. (2004). A meta-analysis of alcohol consumption and the risk of 15 diseases. *Preventive Medicine,* 38 613-619.

Cuijpers, P., Riper, H., & Lemmers, L. (2004). The effects on mortality of brief interventions for problem drinking: a meta-analysis. *Addiction,* 99(7), 839-845.

De Lorenze, G. N., Weisner, C., Tsai, A. L., Satre, D. D., & Quesenberry, C. P. J. (2011). Excess mortality among HIV-infected patients diagnosed with substance use dependence or abuse receiving care in a fully integrated medical care program. *Alcoholism: Clinical and Experimental Research,* 35(2), 203-210.

Harris, E. C., & Barraclough, B. (1998). Excess mortality of mental disorder. *British Journal of Psychiatry,* 173 11-53.

Magill, M., & Ray, L. A. (2009). Cognitive-behavioral treatment with adult alcohol and illicit drug users: ameta-analysis of randomized controlled trials. *J Stud Alcohol Drugs,* 70(4), 516-527.

McQueen, J., Howe, T. E., Allan, L., Mains, D., & Hardy, V. (2011). Brief interventions for heavy alcohol users admitted to general hospital wards. *Cochrane Database of Systematic Reviews,* 8 CD005191.

O'Brien, J. M. J., Lu, B., Ali, N. A., Martin, G. S., Aberegg, S. K., Marsh, C. B. Lemeshow S., & Douglas I.S. (2007). Alcohol dependence is independently associated with sepsis, septic shock, and hospital mortality among adult intensive care unit patients. *Critical Care Medicine,* 35(2), 345-350.

Project MATCH Research Group. (1997). Matching alcoholism treatment to client heterogeneity: Project MATCH posttreatment drinking outcomes. *Journal of Studies on Alcohol,* 58 7-30.

Room, R., Babor, T., & Rehm, J. (2005). Alcohol and public health: a review. *Lancet,* 365 519-530.

Rösner, S., Hackl-Herrwerth, A., Leucht, S., Lehert, P., Vecchi, S., & Soyka, M. (2010a). Acamprosate for alcohol dependence. *Cochrane Database of Systematic Reviews,* 9 CD004332.

Rösner, S., Hackl-Herrwerth, A., Leucht, S., Vecchi, S., Srisurapanont, M., & Soyka, M. (2010b). Opioid antagonists for alcohol dependence. *Cochrane Database of Systematic Reviews,* 12 CD001867.

Smedslund, G., Berg, R. C., Hammerstrom, K. T., Steiro, A., Leiknes, K. A., Dahl, H. M., & Karlsen K. (2011). Motivational interviewing for substance abuse. *Cochrane Database of Systematic Reviews,* 5 CD008063.

Reference List

Corrao, G., Bagnardi, V., Zambon, A. & La Vecchia, C. 2004. A meta-analysis of alcohol consumption and the risk of 15 diseases. *Preventive Medicine,* 38**,** 613-619.

Cuijpers, P., Riper, H. & Lemmers, L. 2004. The effects on mortality of brief interventions for problem drinking: a meta-analysis. *Addiction,* 99**,** 839-845.

De Lorenze, G. N., Weisner, C., Tsai, A. L., Satre, D. D. & Quesenberry, C. P. J. 2011. Excess mortality among HIV-infected patients diagnosed with substance use dependence or abuse receiving care in a fully integrated medical care program. *Alcohol Clin Exp Res,* 35**,** 203-210.

Group, P. M. R. 1997. Matching alcoholism treatment to client heterogeneity: Project MATCH posttreatment drinking outcomes. *Journal of Studies on Alcohol,* 58**,** 7-30.

Harris, E. C. & Barraclough, B. 1998. Excess mortality of mental disorder. *British Journal of Psychiatry,* 173**,** 11-53.

Magill, M. & Ray, L. A. 2009. Cognitive-behavioral treatment with adult alcohol and illicit drug users: a meta-analysis of randomized controlled trials. *Journal of Studies on Alcohol and Drugs,* 70**,** 516-527.

Mcqueen, J., Howe, T. E., Allan, L., Mains, D. & Hardy, V. 2011. Brief interventions for heavy alcohol users admitted to general hospital wards. *Cochrane Database of Systematic Reviews,* 8**,** CD005191.

O'brien, J. M. J., Lu, B., Ali, N. A., Martin, G. S., Aberegg, S. K., Marsh, C. B., Lemeshow, S. & Douglas, I. S. 2007. Alcohol dependence is independently associated with sepsis, septic shock, and hospital mortality among adult intensive care unit patients. *Critical Care Medicine,* 35**,** 345-350.

Room, R., Babor, T. & Rehm, J. 2005. Alcohol and public health: a review. *Lancet,* 365**,** 519-530.

Rösner, S., Hackl-Herrwerth, A., Leucht, S., Lehert, P., Vecchi, S. & Soyka, M. 2010a. Acamprosate for alcohol dependence. *Cochrane Database of Systematic Reviews,* 9**,** CD004332.

Rösner, S., Hackl-Herrwerth, A., Leucht, S., Vecchi, S., Srisurapanont, M. & Soyka, M. 2010b. Opioid antagonists for alcohol dependence. *Cochrane Database of Systematic Reviews,* 12**,** CD001867.

Smedslund, G., Berg, R. C., Hammerstrom, K. T., Steiro, A., Leiknes, K. A., Dahl, H. M. & Karlsen, K. 2011. Motivational interviewing for substance abuse. *Cochrane Database of Systematic Reviews,* 5**,** CD008063.
