# Supplementary material for: Alcohol consumption, alcohol dependence, and related mortality in Italy in 2004: effects of treatment-based interventions on alcohol dependence
Source: Subst Abuse Treat Prev Policy. 2013 Jun 13;8:21. doi: 10.1186/1747-597X-8-21 (PMC3686709; doi:10.1186/1747-597X-8-21)
Supplement: Additional file 3 — Country profile for Italy. [file 1747-597X-8-21-S3.docx]

## Additional file 1: Country profile for Italy

***Country Indicators***

*Size (Size square KM):* ***301,340 (rank: 8)^[[1]](#footnote-1)^***

*Population (2009):* ***62,420,000 (rank: 4)^[[2]](#footnote-2)^***

*GDP PPP per capita (in $US, 2005):* ***28,015 (rank: 15)^[[3]](#footnote-3)^***

***Alcohol Exposure Indicators***

*WHO at risk consumption (2009):* ***W (40+ grams/day): 0.3% M (60+ grams/day): 2.1%***

*Total per capita consumption (unrecorded) [2009]:* ***9.59 litres (l) (2.4 (l) unrecorded)***

Pattern of Drinking Score (2009): **1**

***Alcohol-Attributable Harm (15-64 years of age; 2004)***

*Mortality Alcohol-Attributable (per 100,000):* ***W: 7.57 (rank: 24) M: 18.96 (rank: 24)***

*Percent mortality attributable to alcohol:* ***W: 4.9% (rank: 23) M: 6.3% (rank: 26)***

*DALYs Alcohol-Attributable (per 100,000):* ***W: 146.22 (rank: 28) M: 460.03 (rank: 30)***

*Percent DALYs attributable to alcohol:* ***W: 1.6% (rank: 28) M: 4.5% (rank: 30)***

*Prevalence Alcohol Dependence (18-65):* ***W: 0.85% M: 3.15%***

***Alcohol Dependence Treatment System***

Main psychotherapies: Family-, Group-Therapy, individual counselling; Pharmacotherapies used: disulfiram

**Effects of Interventions**

**Men**

**Women**

**Men**

**Women**

1. The numbers in brackets indicate Italy’s ranking for the respective variable among all 27 EU countries, plus Iceland, Norway and Switzerland. [↑](#footnote-ref-1)
2. These are data from comparable international UN estimates. The local data of residents by ISTAT are lower: 60,626,000 (<http://www.urbistat.it/it/demografia/dati-sintesi/italia/1/1> ) [↑](#footnote-ref-2)
3. Again the local data from ISTAT are slightly different: in 2008 $ 27,982 (<http://www3.istat.it/dati/catalogo/20100409_00/NoiItalia2010.pdf>) [↑](#footnote-ref-3)
